# Case report: Novel *ETFDH* compound heterozygous mutations identified in a patient with late-onset glutaric aciduria type II

**DOI:** 10.3389/fneur.2023.1087421

**Published:** 2023-01-27

**Authors:** Sijia Zhu, Dongxue Ding, Jianhua Jiang, Meirong Liu, Liqiang Yu, Qi Fang

**Affiliations:** ^1^Department of Neurology, The First Affiliated Hospital of Soochow University, Suzhou, China; ^2^Department of General Medicine, The First Affiliated Hospital of Soochow University, Suzhou, China

**Keywords:** glutaric aciduria type II, ETFDH, compound heterozygous mutations, functional analysis, ACMG, riboflavin supplement

## Abstract

Glutaric aciduria type II (GA II) is an autosomal recessive metabolic disorder of fatty acid, amino acid, and choline metabolism. The late-onset form of this disorder is caused by a defect in the mitochondrial electron transfer flavoprotein dehydrogenase or the electron transfer flavoprotein dehydrogenase (*ETFDH*) gene. Thus far, the high clinical heterogeneity of late-onset GA II has brought a great challenge for its diagnosis. In this study, we reported a 21-year-old Chinese man with muscle weakness, vomiting, and severe pain. Muscle biopsy revealed myopathological patterns of lipid storage myopathy, and urine organic acid analyses showed a slight increase in glycolic acid. All the aforementioned results were consistent with GA II. Whole-exome sequencing (WES), followed by bioinformatics and structural analyses, revealed two compound heterozygous missense mutations: c.1034A > G (p.H345R) on exon 9 and c.1448C>A (p.P483Q) on exon 11, which were classified as “likely pathogenic” according to American College of Medical Genetics and Genomics (ACMG). In conclusion, this study described the phenotype and genotype of a patient with late-onset GA II. The two novel mutations in *ETFDH* were found in this case, which further expands the list of mutations found in patients with GA II. Because of the treatability of this disease, GA II should be considered in all patients with muscular symptoms and acute metabolism decompensation such as hypoglycemia and acidosis.

## Introduction

Glutaric aciduria type II (GA II), also called multiple acyl-CoA dehydrogenase deficiency or ethylmalonic-adipic aciduria (MADD; OMIM 231680), is a very rare autosomal recessively inherited disorder with an inborn error of metabolism of amino acid, fatty acid, and choline (1) due to functional defects in either electron transfer flavoprotein (ETF) encoded by the alpha- or beta-subunit ETF gene (*ETFA*, OMIM 608053; *ETFB*, OMIM 130410) or ETF-ubiquinone oxidoreductase (ETF: QO) encoded by the ETF dehydrogenase (*ETFDH*, OMIM 231675) gene (2, 3).

The clinical phenotype of GA II is substantially heterogeneous, from relatively mild late-onset to severe birth defects, including myopathy, cardiomyopathy, pancreatitis, and congenital anomalies (4, 5). It has been categorized into three sub-groups based on these different manifestations: neonatal onset with congenital abnormalities (type 1, OMIM 608053), neonatal onset without abnormalities (type 2, OMIM 231680), and mild and/or later onset (type 3, OMIM 231680) (3, 6, 7). Defects in genes *ETFA, ETFB*, and *ETFDH* are responsible for these three types, respectively (7). Individuals with severe forms always have a rapidly fatal course. Diagnosis of these severe forms mainly relied on the acylcarnitine pattern in dried blood/plasma including short-, medium-, and long-chain acylcarnitine change detected by tandem mass spectrometry (MS/MS) and urine organic acid (UOA) profile (8). However, the milder form, type 3 GA II, caused by the *ETFDH* gene, can present at any age and always show diverse manifestations including fluctuating proximal muscle weakness, intermittent rhabdomyolysis and other nerve neuropathy, weakened or disappeared tendon reflex, and fatty liver (9, 10). Moreover, many patients may present with an episodic illness that poses a great challenge for its diagnosis (11–13). Under these circumstances, molecular studies and genetic testing are required to make a definitive diagnosis.

The therapeutic strategy usually involves a restricted diet including the avoidance of fasting to prevent hypoglycemia and metabolic acidosis, as well as a diet low in protein and fat together with carnitine, ubiquinone, and riboflavin supplement, especially for those with severe forms (14, 15). Patients with milder late-onset forms always have an obvious improvement both in clinical symptoms and metabolic profile with riboflavin supplementation. Recent studies show that these patients, termed riboflavin-responsive MADD (RR-MADD), have been seen to harbor *ETFDH* variants encoding ETF:QO (16, 17).

Herein, we report a 21-year-old male patient who is molecularly confirmed as late-onset GA II. Whole-exome sequencing (WES) revealed a compound heterozygous mutation for two variants in the *ETFDH* gene, namely, a c.1034A>G (p.H345R) in exon 9 and a c.1448C>A (p.P483Q) in exon 11, respectively, located in the FAD-binding domain and UQ-binding domain. In addition, we predict the protein structural changes caused by these mutations, and the results proved these two novel mutations to be very likely pathogenic based both on the clinical findings and protein changes.

## Case report

The patient was a 21-year-old man who came from a non-consanguineous family in Anhui Province. He was admitted to our department due to limb weakness and severe pain accompanied by vomiting for 2 months. In the last 2 weeks, after suffering from influenza, progressive limb weakness reoccurred. He had difficulty walking long distances and climbing the stairs and was too weak to lift his hands to strip or comb the hair. Ten days before admission, he experienced acid reflux, and then repeated nausea and vomiting with gastric contents and bile, with no improvement after fasting and resting up. One week ago, the symptom of limb weakness, pain, and vomiting was noted to have obviously aggravated. Blood test results in the local hospital showed 16,969 u/L creatine kinase (CK) and 2,000 ng/ml myoglobin. Therefore, he was suspected to have gotten rhabdomyolysis in the local hospital and was sent to our department. The patient had normal development during the fetal and infant periods. From 5 years old, he was unable to exercise vigorously because of muscle soreness and aches after activities. Since 2015, he has experienced intermittent acid reflux, nausea, and vomiting; gastroscopy showed chronic superficial gastritis. He had two hospitalizations due to severe vomiting, both occurred in winter. He reported a history of hepatitis carriers for 3 years and denied any history of diabetes and kidney disease. Neurological examination showed limb muscle tenderness, and neck and proximal muscle weakness (manual muscle testing (MMT) score: 4/5 in the neck, 4/5 in upper limbs, and 3/5 in lower limbs).

Besides the elevated level of CK and myoglobin, other parameters were also significantly increased including alanine aminotransferase (ALT) 242 U/L (normal 7–40 U/L), aspartate aminotransferase (AST) 1,745 U/L (normal 13–35 U/L), creatine kinase-MB (CK-MB) 1,425.5 U/L (normal 0–16), myoglobin 2,000 ng/mL (normal 27–70 ng/mL), and lactate dehydrogenase (LDH) 1,813 U/L (normal 120–250 U/L). Urine routine: urine protein (+), urine ketone body (KET) (4+), and urine latent blood (BLD) (3+).

**Figure 1:**
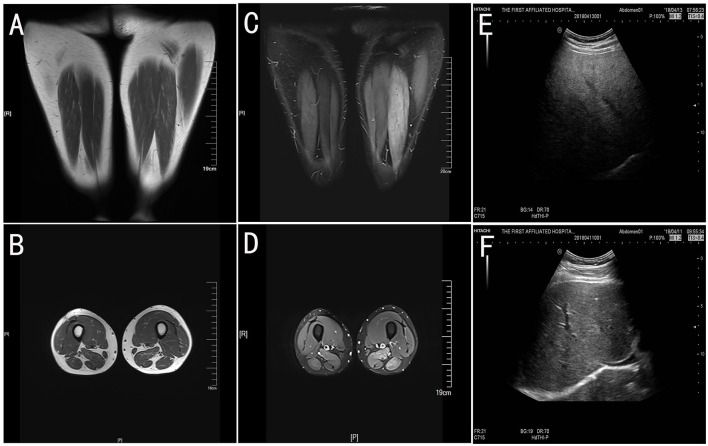
The muscle MRI of lower limbs and abdomen ultrasound. The T1-weight images **(A, B)** showed slightly atrophy of bilateral lower limbs. Fat suppression (T2-weighted short tau inversion recovery, T2-STIR) images **(C, D)** have high signal intensity in the bilateral limb muscles, which indicates muscle injury. The abdomen ultrasound **(E)** showed obvious signs of a fatty liver compared with the health control **(F)**.

Muscle MRI T2 of lower limbs revealed high signal intensity areas in the bilateral lower limb muscles, indicating diffuse muscle injury (Figures 1A–D). Abdomen ultrasound images showed obvious signs of fatty liver (Figure 1E).

**Figure 2:**
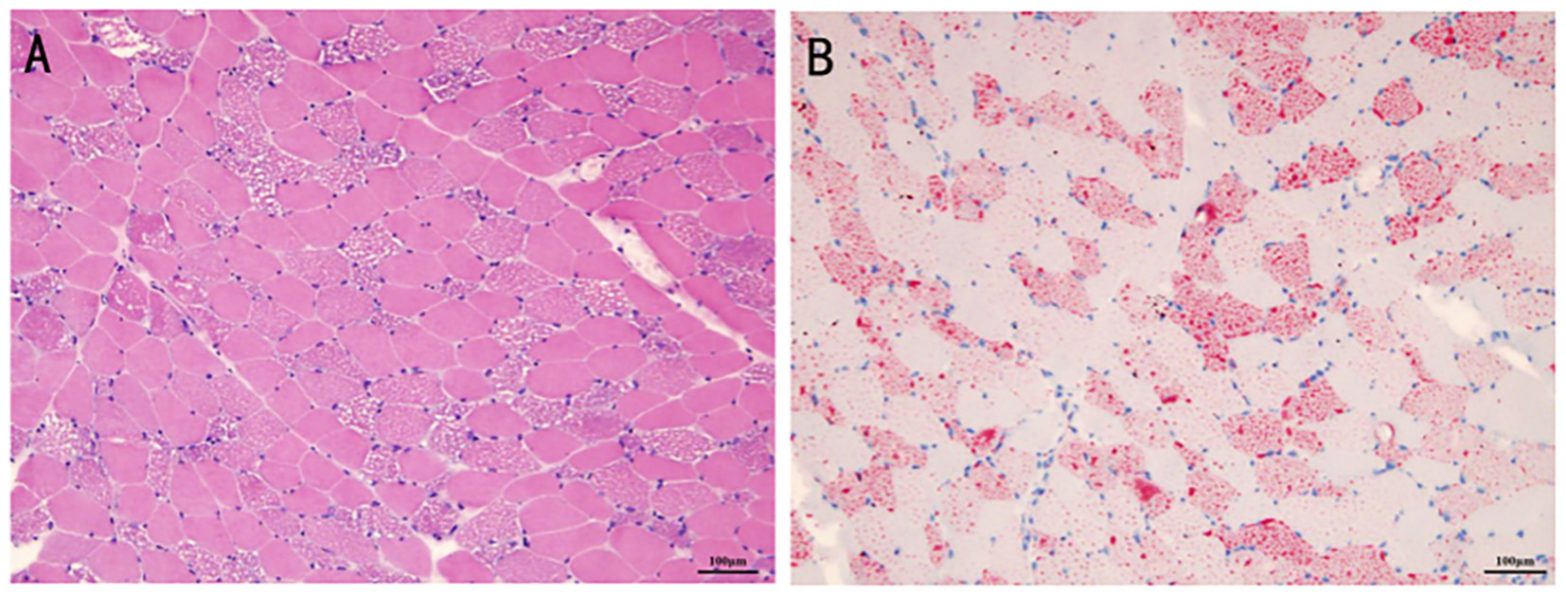
The histopathologic manifestation of the patient's muscle. The formation of vacuoles was found in the muscle fibers for hematoxylin-eosin staining **(A)**. The oil red O staining showed diffuse fat granule aggregation **(B)**.

On admission, he was initially diagnosed with rhabdomyolysis or inflammatory myopathy and was treated with methylprednisolone (40 mg for 2 days, followed by 20 mg for another 2 days). After renal function results returned to normal, the muscle biopsy was performed. The results suggested vacuole myopathy with the formation of vacuoles in muscle fibers (Figures 2A, B). The oil red O staining showed diffuse fat granule aggregation, and periodic acid-Schiff (PAS) staining showed no excessive glycogen content (data not shown). The pathological results were consistent with the manifestation of lipid-storage myopathy. UOA analysis showed that glycolic acid slightly increased to 9.0 (normal 0–8), and no significant increase was observed in the rest results. Blood acylcarnitine analysis showed an increase in octanoyl carnitine (C8) and decanoyl carnitine (C10), while the test results may come after glutaric acid-type II treatment.

**Figure 3:**
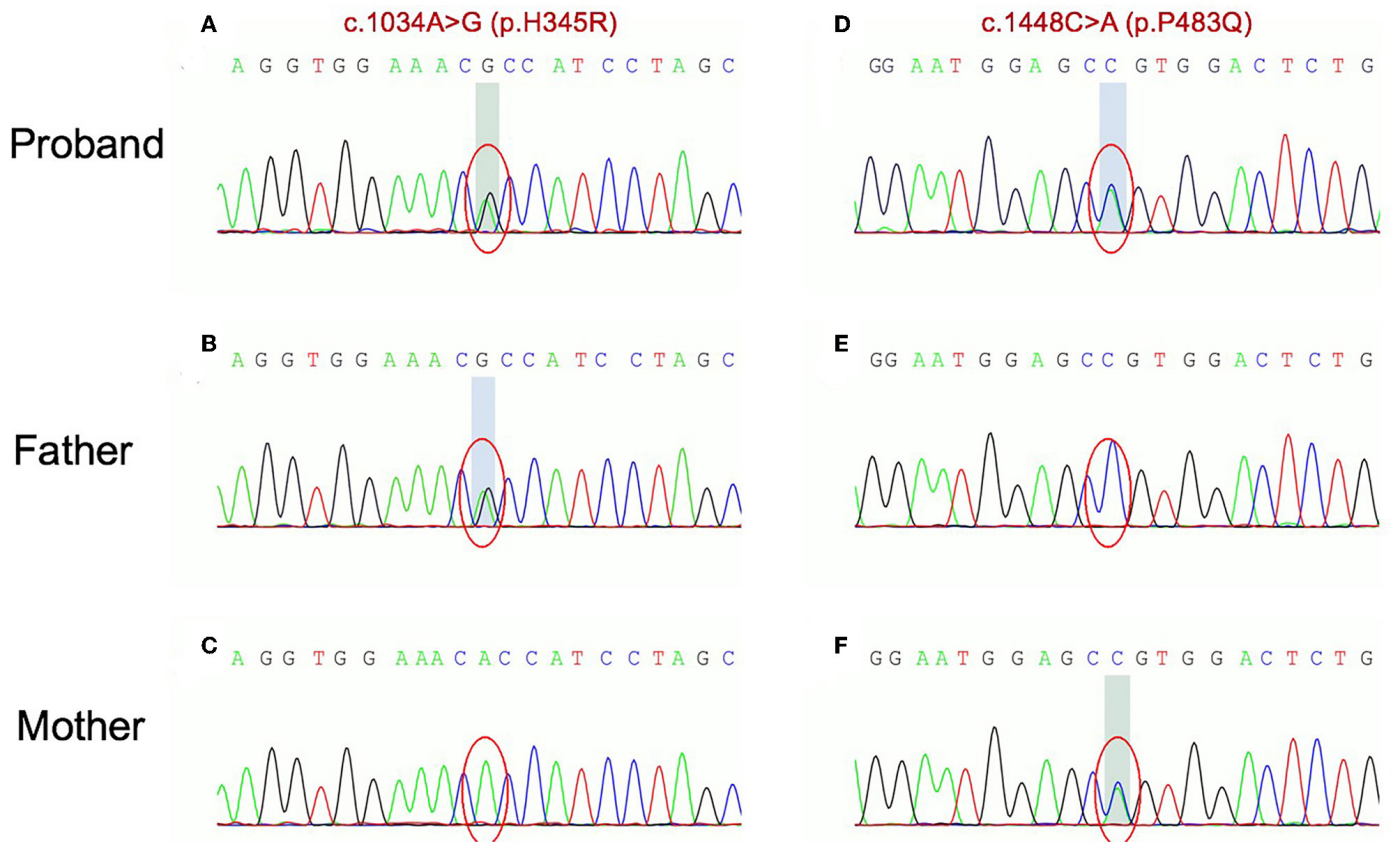
Sanger traces for PCR products of the proband and his parents. Sanger traces for PCR products of the proband indicated a compound heterozygous mutation in ETFDH gene: c.1034A > G (p.H345R) on exon 9 **(A)** and c.1448C>A (p.P483Q) on exon 11 **(B)**. Sanger traces for PCR of his parents. The c.1034A > G (p.H345R) on exon 9 and c.1448C>A (p.P483Q) on exon 11 come from the father and mother **(C–F)**, respectively.

Whole-exome sequencing (WES) revealed a compound heterozygous mutation in the *ETFDH* gene in the proband: c.1034A > G (p.H345R) on exon 9 and c.1448C>A (p.P483Q) on exon 11. Sanger sequencing showed that these two mutations, respectively, come from the proband's father and mother (Figure 3). No mutations were identified in other related genes such as ETFA or ETFB gene.

Both mutations were not reported in the Human Gene Mutation Database and were not found in 100 healthy Chinese control individuals. ETF:QO was shown to have three functional domains: FAD-binding domain, 4Fe−4S cluster domain, and UQ-binding domain. The p.H345R and p.P483Q, respectively, lied in the FAD-binding domain and UQ-binding domain. Bioinformatic and structural analysis was conducted to predict the effect of the revealed variants on the functional properties of the proteins.

**Figure 4:**
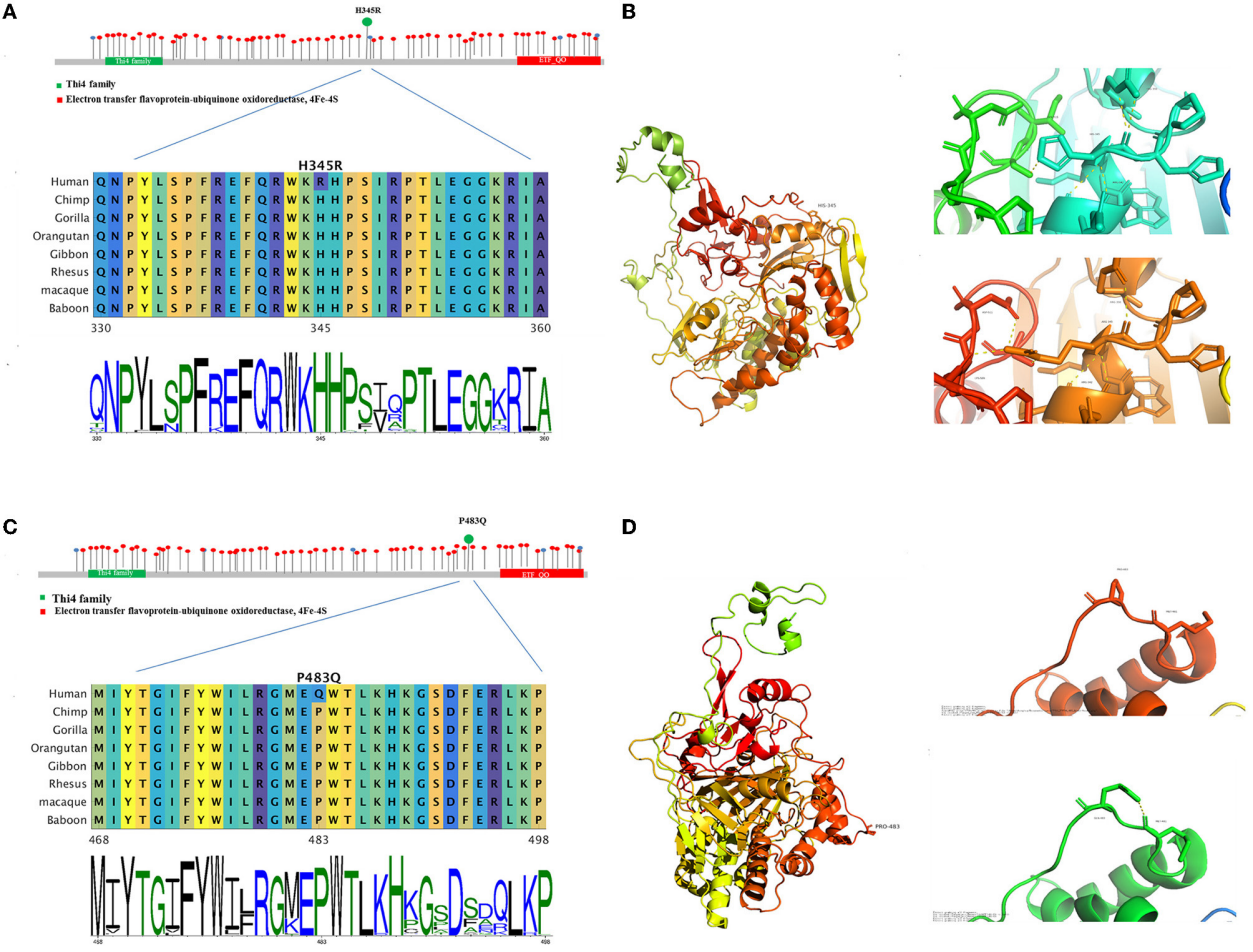
The bioinformatic analysis of the two mutations observed in the proband. Allelic spectrum and location by the functional domain of reported pathogenic mutations of the ETFDH gene were shown. Multiplex sequence alignment of the ETFDH protein across eight different species shows that H345 and P483 are highly conserved among species **(A, C)**. The structure **(B, D)** was built by means of homology modeling based on 2 gmh with Identity 95. The structure is modeled by RosettaCM. The partial 3D structures of both mutations revealed polar interaction changes in the ETFDH protein.

The missense mutation c.1034A > G led to the change from histidine to arginine at 345th amino acid, with no physical and chemical properties change. Multiplex sequence alignment of the *ETFDH* protein across eight different species and 100 vertebrate genomes showed that Histidine (H) at codon 345 was highly conserved among species (Figure 4A). This mutation was graded as variant uncertain significance (VUS) according to ACMG. Results predicted by MutationTaster and SIFT supported that p.H345R of *ETFDH* was a tolerated mutation, while Polyphen-2 identified it as benign. The structural analysis showed a polar interaction change between the wild-type and mutant structures of the *ETFDH* protein (Figure 4B).

The missense mutation c.1448C>A led to the change from proline to glutamine at 483rd amino acid, and the physical and chemical properties change included the size of the side chain change, resulting in the amino acids weight change from 115 to 146. Multiplex sequence alignment also showed that Proline (P) at codon 483 was highly conserved among species (Figure 4C). The mutation was graded as likely pathogenic according to ACMG. Results of Polyphen-2, MutationTaster, and SIFT all supported that p.P483Q of *ETFDH* was a deleterious mutation. The structural analysis revealed a polar interaction change (Figure 4D) and a stability decrease between the wild-type and mutant structures of the *ETFDH* protein.

The patient received a high dose of riboflavin (150 mg/day) with a low-fat diet (lipid restricted to 25% of total calories) after diagnosis. Symptoms of muscle weakness were progressively relieved in 3 weeks and recovered completely in 2 months. In addition, routine blood examinations such as CK, AST, and ALT decreased to normal values at the 6-month follow-up. Until now, the patient remained on riboflavin treatment (150 mg/day) and a low-fat diet. Fortunately, he had no muscle weakness recurrence and was competent for daily life during the 3-year follow-up.

## Discussion

Glutaric aciduria type II is a highly heterogeneous disease characterized by various manifestations with different degrees. RR-MADD, caused by *ETFDH* gene mutations, is the most common phenotype (18). In this study, we described a patient with two novel compound heterozygous mutations in the *ETFDH* gene: c.1034A > G (p.H345R) on exon 9 and c.1448C>A (p.P483Q) on exon 11.

*The electron transfer flavoprotein dehydrogenase* gene is located on chromosome 4q32.1 and consists of 13 exons. To date, more than 200 mutations in the *ETFDH* gene have been identified in Human Gene Mutation Database (HGMD), including various types such as missense, nonsense, insertion, deletion, and splicing mutations (19). The following hot spot mutations were the most frequently identified mutations in Chinese: c.250G > A (p.A84T), c.770A > G (p.Y257C), and c.1227A > C (p.L409F) with a frequency of 12.2%, 15.0%, and 12.2%, respectively (20). Some reports have revealed that the genotype of GA II patients with *ETFDH* mutations was correlated with their diverse phenotype (21). For example, nonsense mutations in both alleles of the *ETFDH* gene resulting in truncation may affect protein structure or stability, which always presents with more severe manifestations. However, the definite correlation between the genotype and phenotype of *ETFDH* mutated late-onset mild GA II has not been fully confirmed due to limited patients and other factors such as infections and poor nutrition. Missaglia described five GA II patients with various *ETFDH* mutations and varying degrees of clinical symptom severity. Similar to our patient, most patients had two compound missense mutations and mainly presented with muscle weakness or exercise intolerance. In addition, our patient had extra-muscle symptoms such as vomiting and fatty liver, while some previously reported patients presented with severe neurological symptoms and metabolic disorders, which further illustrated the high heterogeneity of the disease (22). Therefore, inherited metabolic disease should be considered in clinical work when patients have unexplained myasthenia, exercise intolerance, or other extra-muscle symptoms such as vomiting, seizure, encephalopathy, and hypoglycemia. Blood acylcarnitine analysis and urine organic acid tests could be performed to screen for GA II. Genetic testing should be further conducted to confirm the diagnosis (23).

As a component of the electron-transfer system in mitochondria, the ETF:QO protein forms a short pathway to mediate ATP production by electron transfer from over nine mitochondrial flavin-containing dehydrogenases to the respiratory ubiquinone pool (24–26). It is composed of a 4Fe-4S cluster and one molecule of flavin adenine dinucleotide (FAD) binding domain and the UQ-binding domain (5). It has been reported that regions in the FAD domain could decrease protein stability. While the lack of part of the UQ-binding region and 4Fe4S cluster severely affected the *ETFDH*-mediate electron-transfer pathway (27). The two mutations c.1034A > G (p.H345R) on exon 9 and c.1448C>A (p.P483Q) detected in our study were, respectively, located in the FAD-binding domain and UQ-binding domain. For the first mutation, the structural analysis showed a polar change for the *ETFDH* protein and the second mutation revealed a polar change and stability alteration for this protein. As a previous study reported, *ETFDH* missense variants could cause the misfolding of related proteins, thus inducing structural instability in the protein (28, 29).

According to ACMG guidelines, the pieces of evidence for pathogenic or likely pathogenic variants are graded as four levels: very strong (PVS1), strong (PS1–4), moderate (PM1–6), and supporting (PP1–5) (19). The first mutation affects the amino acid residue 345 causing a substitution of histidine to arginine, putatively affecting the FAD-binding domain. The acid 345H is located in the FAD-binding domain and 15 DMs were located within 30 residues around this residue, indicating that this area is a mutational hot spot related to the disease (PM1). Furthermore, this mutation was absent in Exome Aggregation Consortium (ExAC) or 1,000 Genomes Project (PM2). The multiplex sequence alignment of the *ETFDH* protein across eight different species and 100 vertebrates showed that Histidine (H) at codon 345 in the *ETFDH* gene is highly conserved among species, which indicated that this mutation might have a relatively negative impact. The homology modeling of *ETFDH* protein showed that there were five polar interactions in the wild type, while the mutant H345R had six, which cause the protein structural change. Multiple lines of computational evidence were found to support the deleterious effect on the gene, including the conservation and evolutionary analysis, structural modeling, and computational prediction such as MutationTaster and SIFT (PP3). In this case, the patient's phenotype is highly specific for GA II (PP4). Based on these findings, it could be classified as “likely pathogenic” according to ACMG (2 PM + 2 PP).

The second mutation affects the amino acid residue 483 causing proline to glutamine. The acid 483P is located in the UQ-binding domain and 19 DMs were located within 30 residues around P483, also indicating a mutational hot spot (PM1). This mutation was not found in ExAC or 1000 Genomes Project (PM2). In the ClinVar database, a different mutation c.1448C>T (p.P483L) at the same amino acid was reported to be “likely pathogenic” (PM5). Furthermore, the Proline (P) at codon 483 is also highly conserved among species. The homology modeling of the *ETFDH* protein showed that there was no polar interaction in the wild type, while the mutant P483Q had one, which could cause the protein structural change. Furthermore, we found that the 483rd Proline mutated to other 19 amino acids could cause a significant rise of the structural folding free energy, resulting in a decline of thermodynamic stability. The deleterious effect was supported by the conservation and evolutionary analysis, structural modeling, and computational prediction including MutationTaster, Polyphen2, and SIFT (PP3). Considering the patient's phenotype (PP4), this mutation should be classified as “likely pathogenic” (3 PM + 2 PP).

Similar to most patients with late-onset GA II, our patient showed a good response to riboflavin treatment. Riboflavin, commonly known as vitamin B2, is the precursor of flavin cofactors, which exists extensively in our daily food and is metabolized to (FMN) and FAD (30). Thus, the role of riboflavin in stabilizing certain forms of ETF: QO variant proteins is undoubted. The common view supports long-term, high-dose therapy of riboflavin. However, a recent study showed that most patients did not need continuous high-dose riboflavin treatment. Patients had a low risk of recurrence with intermittent and low-dose riboflavin treatment. Some patients even remained asymptomatic after discontinuation of riboflavin. A hypothesis is that riboflavin increased FAD-binding flavoproteins, which could release more FAD to the mitochondrial matrix during degradation, remaining a larger circulating FAD pool even after discontinuation of riboflavin (5). In addition to riboflavin treatment, a low-fat, low-protein, and high-carbohydrate diet should be provided to reduce metabolic disorders and ensure an adequate energy supply. L-carnitine and coenzyme Q10 can also be given to patients with carnitine deficiency and coenzyme Q10 deficiency, respectively (31). Since the high clinical heterogeneity and treatability of late-onset GA II, screening the hot spot mutations in Chinese patients clinically suspected of GA II could be beneficial for its early diagnosis and treatment, during which the WES strategy would be a great tool to identify novel pathogenic spots.

## Conclusion

In summary, we described the phenotype and genotype of a patient with late-onset GA II. Two novel likely pathogenic mutations, c.1034A > G (p.H345R) and c.1448C>A (p.P483Q) mutations were identified, enriching the spectrum of *ETFDH* mutations associated with GA II. Due to its atypical symptoms and its good responsibility to riboflavin of late-onset mild GA II, the precise diagnosis is of great importance, and genetic testing would be useful to avoid misdiagnosis and missed diagnosis.

## Data availability statement

The original contributions presented in the study are included in the article/supplementary material, further inquiries can be directed to the corresponding authors.

## Ethics statement

The studies involving human participants were reviewed and approved by the First Affiliated Hospital of Soochow University. The patients/participants provided their written informed consent to participate in this study. Written informed consent was obtained from the individual(s) for the publication of any potentially identifiable images or data included in this article.

## Author contributions

SZ and DD conducted the case series and drafted the manuscript. SZ, DD, and JJ participated in the collection and collation of clinical data. ML conducted the muscle biopsy and pathological analysis. LY and DD were responsible for data analysis of genetic testing and Sanger sequencing. LY and QF were involved in revising the manuscript critically and have given final approval for the version to be published. All authors have read and approved the manuscript.
